# Voltammetric Determination of Salbutamol, Sulfamethoxazole, and Trimethoprim as Anthropogenic Impact Indicators Using Commercial Screen-Printed Electrodes

**DOI:** 10.3390/s25102998

**Published:** 2025-05-09

**Authors:** Jing Huang, Julio Bastos-Arrieta, Núria Serrano, José Manuel Díaz-Cruz

**Affiliations:** 1 Department of Chemical Engineering and Analytical Chemistry, Universitat de Barcelona (UB), Martí i Franquès 1-11, 08028 Barcelona, Spain; jing.huang@ub.edu (J.H.); nuria.serrano@ub.edu (N.S.); 2 Water Research Institute (IdRA), University of Barcelona (UB), Martí i Franquès 1-11, 08028-Barcelona, Spain

**Keywords:** emerging contaminants, differential pulse voltammetry, carbon screen-printed electrodes, salbutamol, sulfamethoxazole, trimethoprim

## Abstract

A voltammetric method based on the use of screen-printed carbon electrodes (SPCEs) is presented for the simultaneous determination of salbutamol (SAL), sulfamethoxazole (SMX), and trimethoprim (TMP), with high sensitivity, fast response, and excellent repeatability and reproducibility. Under the optimal voltammetric conditions, the simultaneous analysis showed linear ranges of 0.3–2.5 mg L^−1^, 0.3–11.1 mg L^−1^, and 0.5–9.0 mg L^−1^ for SAL, SMX, and TMP, respectively, and detection limits of 83.8 μg L^−1^, 88.7 μg L^−1^, and 139.2 μg L^−1^, respectively. Additionally, the developed method was successfully validated by the analysis of a spiked river water sample with satisfactory recovery values in the range of 97.0–98.8%. The added value of the presented method relays in combining cost-effective disposable SPCEs with rapid analysis (<30 s), providing portable electrochemical tools for the on-site monitoring of pharmaceutical residues, which is critical for addressing contamination linked to anthropogenic activity.

## 1. Introduction

Since the Industrial Revolution, increasing human activity has contributed to global climate change. Climate change affects human health through complex pathways, including the spread and prevalence of disease through the destruction of clean air, safe drinking water, and food supplies. As noted by the World Health Organization (WHO), global warming affects human health, with a notable impact on respiratory (allergy, asthma, bronchitis, and chronic obstructive pulmonary disease) and cardiovascular diseases (an increase in cardiac emergencies during heatwaves, dehydration, and heart problems), as well as mental health. Therefore, the significant impact of climate change on public health has led to an increase in the demand for pharmaceuticals worldwide [1]. These pharmaceuticals, used not only on humans but also on animals and plants, are entering the environment and water sources through wastewater, waste, run-off, and sewage, thus contributing to the emergence and spread of drug-resistant bacteria, such as ‘superbugs’ [2]. Pharmaceutical residues in the environment are considered an important group of emerging contaminants (ECs) because of their non-natural degradability and their potentially harmful effects on aquatic animals and humans through the water cycle [3]. Therefore, it is critical and relevant to propose simple, inexpensive, rapid, sensitive, and selective analytical methodologies for the detection of these types of ECs in environmental samples.

Conventional analytical techniques such as spectrophotometry [4], high-performance liquid chromatography–mass spectrometry (HPLC-MS) [5], and gas chromatography–mass spectrometry (GC-MS) [6] are a good choice for the accurate determination of ECs, but they face limitations such as equipment cost, complex operation procedures, and limited portability. In recent years, electrochemical analysis technology has not only shown excellent sensitivity and selectivity but also presents the advantages of cost-friendliness, facility of operation, rapid analysis, and suitable on-site detection [7]. Among them, voltammetric methods are the most widely used electrochemical techniques for the determination of ECs in various complex environmental matrices due to their high accuracy and sensitivity [8,9,10].

Electrochemical sensors based on voltammetric techniques have been extensively used for monitoring pharmaceutical residues in environmental samples [11,12,13,14]. Compared with other sensing tools, screen-printed carbon electrodes (SPECs) are suitable electrochemical sensors for in situ environmental analysis due to their portability, low power consumption, and fast response. Moreover, the working electrodic surface of SPCEs can be modified to accommodate specific contaminants and improve their electroanalytical performance [15,16]. However, due to the overlapping signals of multiple pharmaceutical analytes in similar potential ranges, voltammetry coupled with SPCEs has been scarcely applied for the simultaneous determination of multiple pharmaceutical residues in environmental samples. Our group proposed a fast and cost-effective voltammetric method for the simultaneous determination of paracetamol, ibuprofen, and caffeine (with detection limits of 0.03, 0.6, and 0.05 mg L^−1^, respectively) in water samples using commercial SPEs based on carbon nanomaterials [17]. More recently, a voltammetric method based on the use of SPCEs was also proposed for the simultaneous determination of ascorbic acid, paracetamol, dextromethorphan, and caffeine in both pharmaceutical products and spiked well water samples, achieving detection limits of 0.5, 0.2, 0.3, and 0.5 mg L^−1^, respectively [18]. Moreover, a chemometric survey about the ability of voltammetry to discriminate seven characteristic pharmaceutical products from the evolution of signals as a function of pH was performed, providing a feasible strategy for the qualitative sensing of pharmaceuticals in natural waters and wastewaters [19].

Salbutamol (SAL), also known as albuterol and sold under the brand name Ventolin, among others, is a bronchodilator used to relieve symptoms of asthma and chronic obstructive pulmonary disease such as wheezing, coughing, and feeling breathless [20]. Sulfamethoxazole (SMX) and trimethoprim (TMP) are typically combined in antimicrobial formulations used to treat and prevent many bacterial infections (such as middle ear, intestinal, urine, and respiratory infections) and to prevent and treat a certain type of pneumonia (pneumocystis-type) [21]. Therefore, SAL, SMX, and TMP are three common clinical therapeutic pharmaceuticals that can be considered as anthropogenic impact indicators and targeted analytes in environmental analysis. Likewise, uric acid (UAC) is a natural waste product of the metabolic breakdown of purines that frequently coexist with pharmaceuticals in biological and environmental samples [22].

Accordingly, in this work, we present the development and optimization of a voltammetric method using a commercial SPCE based on a three-electrode configuration for the determination of UAC, SAL, SMX, and TMP. The optimized experimental conditions ensured the appropriate identification and quantification of the pharmaceutical residues with suitable analytical performance, including their determination in real surface water samples.

## 2. Materials and Methods

### 2.1. Chemicals and Materials

Uric acid (UAC), salbutamol (SAL), sulfamethoxazole (SMX), and sodium hydrogen phosphate were provided by Sigma-Aldrich (Burlington, MA, USA). Trimethoprim 98% (TMP) was purchased from Thermo Fisher Scientific (Waltham, MA, USA). Potassium dihydrogen phosphate and boric acid were provided by PanReac AppliChem (Castellar del Vallès, Spain). Tris(hydroxymethyl)aminomethane (Tris), acetic acid, sodium chloride, potassium chloride, potassium hydroxide, hydrochloric acid, and phosphoric acid were purchased from Merck KGaA (Darmstadt, Germany). Sodium hydroxide was provided by Scharlau Chemie (Setmenat, Spain). All reagents used were of analytical grade.

Stock solutions of approximately 1000 mg L^−1^ of UAC, 1300 mg L^−1^ of SAL, 1400 mg L^−1^ of SMX, and 1600 mg L^−1^ of TMP were prepared weekly to prevent changes in their concentration. The UAC and SAL stock solutions were prepared in ultrapure water obtained from a Milli-Q reference A+ System (Millipore, Molsheim, France). The stock solution of SMX was prepared in ultrapure water by adding 0.1 mol L^−1^ NaOH dropwise until it was completely dissolved. The stock solution of TMP was prepared by adding 0.1 mol L^−1^ HCl dropwise until complete dissolution. The corresponding daily working concentration solutions were prepared with appropriate dilution in ultrapure water from stock solutions and stored in the refrigerator at 4 °C.

Britton–Robinson (BR) aqueous universal buffer solutions were prepared in a pH range from 4 to 10, composed of a mixture of acetic acid, boric acid, and phosphoric acid at a concentration of 0.04 mol L^−1^ each, and adjusted to the desired pH with 0.1 mol·L^−1^ KOH. Similarly, 0.1 mol L^−1^ solutions of phosphate-buffered saline (PBS) were prepared in a pH range from 6 to 8, adjustingthe desired pH with 0.1 mol L^−1^ HCl. In the case of 0.1 mol L^−1^ Tris(hydroxymethyl)aminomethane hydrochloride aqueous universal buffer solutions (Tris-HCl), these were prepared in a pH range from 7 to 9, adjusting the pH with 0.1 mol L^−1^ HCl.

The river water sample was collected in the Ripoll River (Sabadell, Barcelona, Spain) and used for the evaluation of the suitability of the developed method for the analysis of the target pharmaceutical residues.

### 2.2. Instrumentation

Differential pulse voltammetric (DPV) analyses were carried out using a screen-printed carbon electrode (SPCE) with 4 mm diameter (ref. 110) (Metrohm DropSens, Oviedo, Spain), which is connected by means of a flexible cable (ref. CAC) (Metrohm DropSens) to a PGSTAT 204 Autolab potentiostat (EcoChemie, Utrecht, The Netherlands), controlled with the software Nova 2.1.5 (Ecochemie). In DPV measurements, the solution under study was placed on a 10 mL glass vessel, and the stirring was performed using an external magnetic stirrer supplied by IKA (Staufen, Germany). For pH control, a Crison micropH 2000 (Hach Lange, Loveland, CO, USA) was used.

### 2.3. Differential Pulse Voltammetric Measurements

DPV measurements were performed by scanning the potential from 0 V to 1.3 V using a modulation amplitude of 0.1 V, a step potential of 5 mV, a modulation time of 50 ms, and a scan rate of 0.01 V s^−1^. A preconditioning potential of −0.5 V for 30 s is required to remove oxidized species from the SPCE surface. The set of DPV parameters was optimized in previous studies that showed good results for different pharmaceutical residues, SMX among them [18,23]. All experiments were carried out without any oxygen removal. Before starting every assay, repeated measurements of the blank were recorded in the appropriate buffer solution until a stable background current was achieved.

Individual and simultaneous calibration experiments for UAC, SAL, SMX, and TMP were conducted, unless stated otherwise, in 0.1 mol L^−1^ Tris-HCl buffer at pH 7, progressively increasing the concentrations of the target compounds. Each determination was performed in triplicate following an external calibration approach, employing a new SPCE for each replicate.

For the quantification of SAL, SMX, and TMP in water samples from the Ripoll River, the collected samples were fortified with 0.96 mg L^−1^ of SAL, 1.02 mg L^−1^ of SMX, and 1.19 mg L^−1^ of TMP. In the DPV measurements, a volume of the spiked river water sample was mixed with an equal volume of Tris-HCl buffer at pH 7, resulting in a 1:2 dilution factor. Following the initial voltammogram acquisition, four sequential additions of standard solutions containing SAL, SMX, and TMP were carried out, and the corresponding DPV curves were recorded. The analyses were carried out in triplicate using the standard addition method, using a new SPCE for each replicate [24].

## 3. Results and Discussion

### 3.1. pH Optimization

The pH value of the media is of great significance to the measurement of oxidation peaks since protons are involved in the corresponding electrode reaction (see proposed oxidation mechanisms in Supplementary Information Figure S1) [19]. Oxidation potential change may occur over a pH range. These shifts are attributed to proton-coupled electron transfer mechanisms, wherein the protonation state of each compound, controlled by the solution pH [19], influences the potential required for oxidation. The electrochemical profiles reflect changes in molecular speciation, particularly for ionizable functional groups such as phenols (in SAL), sulfonamides (in SMX), and amines (in TMP). This pH-dependent behavior emphasizes the importance of controlling and understanding pH conditions in electroanalytical detection.

**Figure 1:**
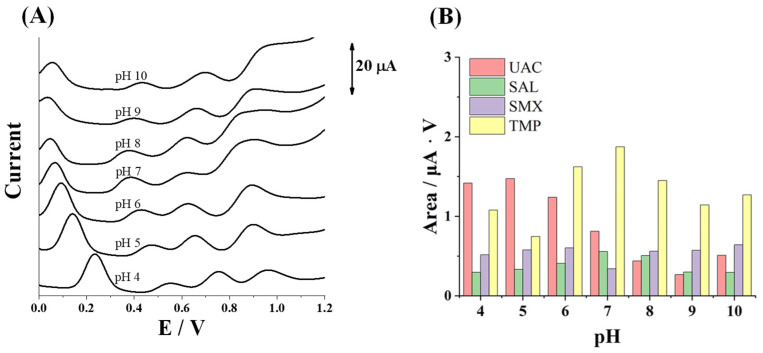
(**A**) Simultaneous measurements of 25.0 mg L^−1^ UAC, SAL, SMX, and TMP by DPV in Britton–Robinson buffer solution at different pH values. (**B**) Plot of the oxidation peak area of each analyte at different pH values.

The influence of pH on the electrochemical responses of SPCE towards the simultaneous determination of UAC, SAL, SMX, and TMP is shown in Figure 1. Measurements of a solution containing 25.0 mg L^−1^ UAC, SAL, SMX, and TMP were carried out in BR buffer solutions at pH values ranging from 4 to 10. The pH effect on individual measurements of the analytes can be seen in the Supplementary Information Figure S2. Figure 1B shows more clearly the evolution of the peak area of each analyte at the studied pH values. As can be seen, the oxidation peak area of SAL and TMP reached a maximum at pH 7, whereas the oxidation peak area of UAC and SMX reached a maximum at pH 5 and pH 6, respectively.

Then, the next step was the optimization of the buffer solution. For this purpose, three different buffer solutions (Tris-HCl, BR, and PBS) within the effective buffer range were chosen to investigate their effect on the electrochemical responses to the simultaneous determination of UAC, SAL, SMX, and TMP. Specifically, the tested buffer solutions were BR (pH 6 to 8), 0.1 mol L^−1^ PBS (pH 6 to 7), and 0.1 mol L^−1^ Tris-HCl (pH 7 to 9). The simultaneous measurements of the considered analytes in different buffers in a pH range from 6 to 9 are supplied in Figure S3. As can be seen, for all considered analytes, the most well-defined and separated signals were achieved using Tris-HCl buffer, particularly at pH 7 (Figure S3B). Therefore, for further measurements, Tris-HCl buffer at pH 7 was selected as the optimal medium, showing similar results to the reported individual [25,26,27,28] or simultaneous (SMX and TMP) [21,29] measurements.

### 3.2. Analytical Performance Evaluation

**Figure 2:**
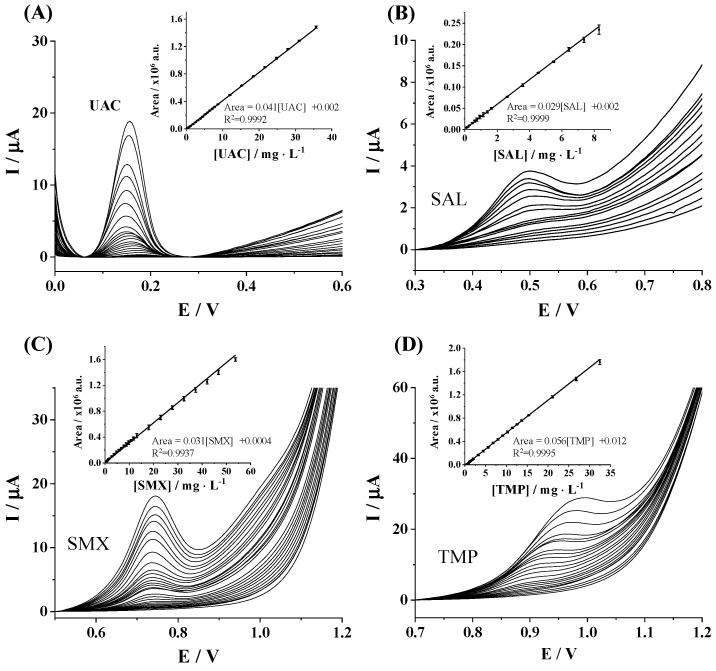
Individual DPV responses of (**A**) UAC, (**B**) SAL, (**C**) SMX, and (**D**) TMP; and their respective calibration plots (insets) in 0.1 mol L^−1^ Tris-HCl buffer (pH 7) on SPCE. The error bars in the calibration plots represent the standard deviations from replicate calibration curves carried out with three different SPCE units.

The electrochemical performance of the SPCE for the individual determination of UAC, SAL, SMX, and TMP was evaluated by determining the sensitivity, R^2^, linear range, limit of detection (LOD), and limit of quantification (LOQ). Firstly, DPV current responses of individual target pharmaceuticals were recorded at increasing concentrations, namely, 0.07–35.7 mg L^−1^, 0.01–8.3 mg L^−1^, 0.1–57.3 mg L^−1^, and 0.2–32.5 mg L^−1^ for UAC, SAL, SMX, and TMP, respectively. As can be seen in Figure 2A–D, well-defined oxidation peaks over the analyzed concentration ranges were obtained for all considered pharmaceutical compounds. Good linear responses of peak area *vs.* target compound concentration were achieved for all tested pharmaceutical compounds (Figure 2A–D insets). The analytical performance for the individual determination of target analytes is summarized in Table 1. Sensitivity was computed as the slope of the regression line. LOD and LOQ values were calculated by using International Union of Pure and Applied Chemistry (IUPAC) definitions, considering LOD as 3S_a_/b and LOQ = 10 S_a_/b, where S_a_ is the standard deviation of the intercept and b is the slope of the calibration straight line in the considered concentration range. The linear range is given between the achieved LOQ and the upper concentration for which suitable linearity has been demonstrated. As can be observed from Table 1, LOD values are in the range of 67.0 to 235.1 µg L^−1^ depending on the studied pharmaceutical compound, and linear responses up to 35.7, 8.3, 53.8, and 32.5 µg L^−1^ were attained for UAC, SAL, SMX, and TMP, respectively. Common concentrations of these analytes in environmental water sources are in the lower range of 1–250 µg·L^−1^, reaching values up to 1000 µg L^−1^ for SAL [30,31].

**Figure 3:**
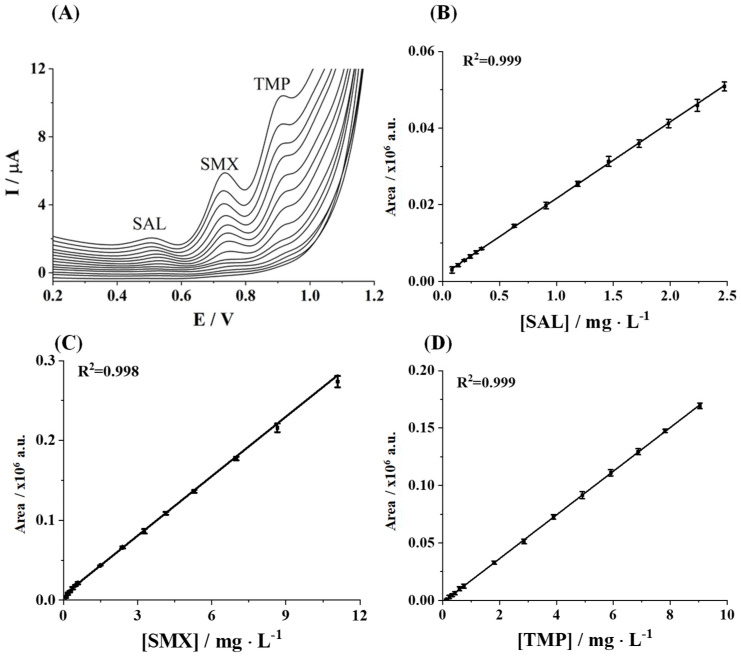
(**A**) Simultaneous DPV responses of SAL, SMX, and TMP at increasing concentrations and their calibration plots in 0.1 mol L^−1^ Tris-HCl buffer (pH 7) on SPCE: (**B**) SAL, (**C**) SMX, and (**D**) TMP. The error bars in the calibration plots represent the standard deviations from replicate calibration curves carried out with three different SPCEs.

Consequently, the simultaneous voltammetric determination of UAC, SAL, SMX, and TMP was attempted, limiting the concentrations of each analyte to the ranges for which the linear calibration curves were obtained in the individual assay. It is worth noting that, at lower concentrations, the area of the oxidation peak of UAC is relatively small. Moreover, it was observed that UAC lost linearity in simultaneous determination, overlapping with the SAL peak as concentration increased (Figure S4). This fact can be attributed to the competition between analytes for the working electrode surface. The SAL benzene ring and the UAC purine ring exhibit adsorption affinity toward hydrophobic domains of screen-printed carbon electrodes, and the oxidation reaction may occur in the same or adjacent potential range [32,33]. Besides this, the electron transfer kinetics at unmodified SPEs can lead to peak broadening, exacerbating voltammetric overlap. Higher analyte concentrations may induce overlapping diffusion layers between UA and SAL current responses. Under these conditions, the oxidation peaks may appear as a single broadened peak rather than distinct dual peaks. Consequently, SAL, SMX, and TMP were finally considered as target pharmaceuticals for simultaneous voltammetric determination. As shown in Figure 3A, three clear, defined, and well-separated peaks were observed at potentials around 0.51 V, 0.74 V, and 0.92 V for SAL, SMX, and TMP, respectively, which were similar to those obtained in the individual determination. Their respective calibration plots are illustrated in Figure 3B–D, providing good linear responses of the peak area as a function of the concentration of the studied analytes.

The analytical performance for the simultaneous determination of SAL, SMX, and TMP can be seen in Table 1. Compared to the calibration data achieved in the individual determination of SAL, SMX, and TMP, it can be observed that lower sensitivities were obtained for all the considered compounds, resulting in narrower linear ranges (linear responses up to 2.5, 11.1, and 9.0 µg L^−1^, respectively).

This can be explained in terms of the competitiveness of compounds to react in the electrode surface. However, lower LODs were achieved in the simultaneous calibration (from 83.8 to 139.2 μg L^−1^), indicating that such competitive behavior is not seriously affecting the performance of the proposed methodology. In comparison with previously reported works, summarized in Table 2, the achieved LOD values are comparable or even better, considering that the lowest LODs have been reported for single analytes using electrodes with sophisticated modifications (including conductive polymers, nanomaterials, and reduced graphene oxide) and mostly on glassy carbon electrodes (GCEs). The versatility, cost-effectiveness, and portability of the bare SPCE result in an advantageous added value consideration of the developed method, which is also supported by electrochemical impedance spectroscopy characterization (details in Supplementary Information and Figure S5).

Repeatability and reproducibility calculation was carried out to evaluate the accuracy of the proposed voltammetric method. Accordingly, DPV measurements in a solution containing 25.0 mg L^−1^ SAL, SMX, and TMP were carried out at the optimal conditions. The repeatability was obtained from the relative standard deviation (RSD, %) of ten successive measurements in the above-mentioned mixture solution using a single SPCE unit. Similarly, three SPCE units were tested under the same conditions to evaluate the reproducibility. As shown in Table 3, RSD ranged from 1.3 to 3.5% and from 2.8 to 4.3% for repeatability and reproducibility, respectively, depending on the studied pharmaceutical compound.

These values are similar to those obtained in previous works for the voltammetric determination of pharmaceutical residues, which indicate that the repeatability and reproducibility of the proposed method are suitable [37,38,39,40,65,66]. In this sense, the applicability of the developed voltammetric method has an added value on the fact that is based on the use of a non-modified and commercially available SPCE (scarcely used in the literature). It is worth highlighting that, to our best knowledge, the simultaneous determination of SAL, SMX, and TMP has not been previously reported.

Moreover, from the obtained results, it can be concluded that the use of an unmodified SPCE for the simultaneous determination of SAL, SMX, and TMP could be perfectly suitable at concentration levels usually found in polluted environmental samples and wastewater (see Supplementary Information regarding interference, stability, and reproducibility assays, Figures S6 and S7). In addition, it should be highlighted that bare SPCE is a low-cost, portable, non-toxic, and reproducible device that does not need any cleaning, polishing, or modification procedure, considerably reducing the time devoted to the preparation of the sensor.

### 3.3. Application to a Real Sample

**Figure 4:**
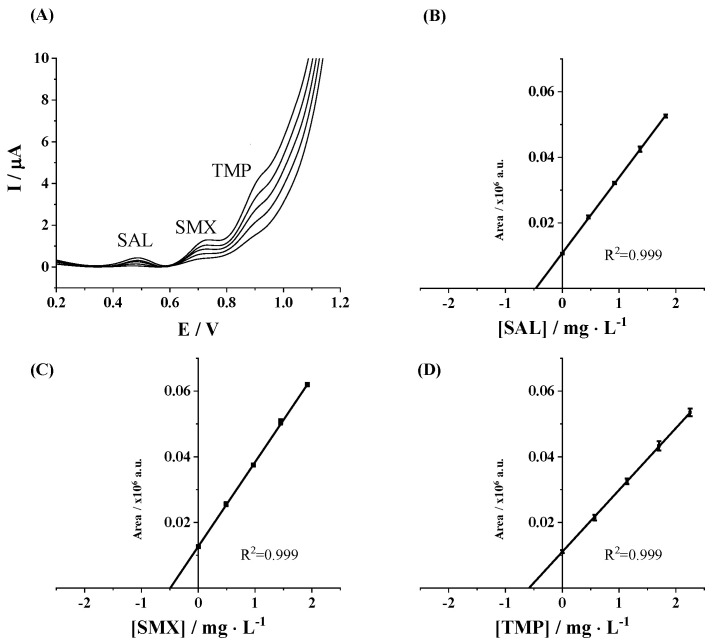
(**A**) DPV measurements in a spiked river water sample in 0.1 mol L^−1^ Tris-HCl buffer (pH 7) on SPCE. (**B**) SAL, (**C**) SMX, and (**D**) TMP standard addition plots.

The applicability of the developed method was validated by using it to determine the concentration of the considered pharmaceutical residues in a spiked real water sample, such as river water (Ripoll River, Barcelona, Spain). Thus, the simultaneous determination of pharmaceutical residues by DPV using an SPCE was performed in triplicate in the spiked river water sample as indicated in the Section 2. As displayed in Figure 4A, the voltammograms obtained from SAL, SMX, and TMP by the standard addition method showed well-defined peaks and similar behavior to those obtained for the simultaneous calibration of pharmaceuticals. A good correlation of the DPV measurements was achieved as illustrated in the SAL, SMX, and TMP standard addition calibration plots (Figure 4B–D). As can be seen in Table 4, very good recovery values (>97.0%) of the spiked river sample and a good agreement between SAL, SMX, and TMP concentration from the three analyses (RSD values <3.3%) were achieved. Thus, these results indicate that the developed method, based on a simple commercial SPCE, is validated and reliable for determining targeted pharmaceutical residues in environmental water samples.

Maximum permissible concentrations (MPCs) for SAL and TMP are not explicitly defined in many environmental regulations, as they are classified as emerging contaminants. Studies have suggested a protective concentration of 100 µg·L^−1^ for TMP to mitigate ecological risks and the promotion of antibiotic resistance [67]. Regarding SMX, the concentration limit in tap water has been reported as 2 × 10^−7^ mol·L^−1^ (~40 µg·L^−1^) [68], though concentrations in river water are typically much lower. Highly sensitive methods capable of detecting SAL in the ng·L^−1^ range have been reported [69], with more complex and costly instrumentation. SAL, SMX, and TMP are partially degraded in wastewater treatment processes, leading to extremely low environmental levels (ng·L^−1^ range). However, the primary aim of this study was to develop and optimize a voltammetric method using a bare screen-printed carbon electrode (SPCE) as a baseline platform, which could be a suitable analytical control early-warning tool for situations where these compounds are present at elevated concentrations, particularly in the micromolar range, for instance, near pollution sources or in cases in which water treatment plants fail. Improved sensitivity could be achieved by the surface modification of the SPCE, including polymeric coating to increase the preconcentration of the analytes through electrostatic or specific interactions, especially considering the analyte charge at different pH levels. Additionally, the incorporation of nanomaterials such as carbon nanotubes or metal oxides could significantly enhance electrocatalytic activity and sensitivity.

## 4. Conclusions

DPV coupled with SPCE was used for the first time for the simultaneous voltammetric determination of UAC, SAL, SMX, and TMP. pH and buffer solutions were first optimized and 0.1 mol L^−1^ Tris-HCl buffer at pH 7 was the one with the optimal DPV response. Then, analytical performance was assessed for both individual and simultaneous determinations of the considered pharmaceuticals. Excellent DPV responses and respective calibration plots were obtained for all compounds in individual calibrations. In addition, the simultaneous analysis presented three well-defined voltammetric peaks. The linear ranges obtained for the simultaneous determination of SAL, SMX, and TMP were slightly narrower than those achieved by individual determination, with low LODs (SAL: 83.8 μg L^−1^; SMX: 88.7 μg L^−1^; TMP: 139.2 μg L^−1^). Finally, the low-cost and reproducible voltammetric method using disposable electrodes was applied to the determination of SAL, SMX, and TMP in spiked river water to investigate its applicability to environmental samples, and satisfactory results were obtained.

The studied analytes are frequently detected in wastewater and surface waters due to their widespread use in human and veterinary medicine. Their persistence and mobility make them effective anthropogenic markers for monitoring urban wastewater contamination in aquatic environments. SMX and TMP have limited biodegradability, making their presence in environmental waters proof of untreated or poorly treated sewage effluents. SAL presents higher biodegradability that can be detected in water bodies, particularly near high-population-density areas. The implementation of the developed voltammetric method for the detection of these pharmaceuticals would facilitate the tracing of the sources and magnitude of anthropogenic pollution and the assessment of the efficiency of wastewater treatment plants. Consequently, in our view, the present study is another step toward providing disposable, commercial, and cost-effective electroanalytical tools for the screening and on-site monitoring of pharmaceutical residues in natural and wastewater samples.

## Figures and Tables

**Table 1:** Analytical performance for the individual (Ind.) and simultaneous (Sim.) determination of target analytes on SPCE in 0.1 mol L^−1^ Tris-HCl buffer (pH 7). The standard deviations are denoted by parentheses.

	UAC		SAL		SMX		TMP	
	Ind.	Sim.	Ind.	Sim.	Ind.	Sim.	Ind.	Sim.
Sensitivity (nA V mg^−1^ L)	1.17 (0.01)	-	1.62 (0.08)	1.14 (0.04)	1.95 (0.04)	1.59 (0.04)	4.89 (0.07)	1.16 (0.02)
Linear range (mg L^−1^)	0.2–35.7	-	0.3–8.3	0.3–2.5	0.4–53.8	0.3–11.1	0.8–32.5	0.5–9.0
R^2^	0.999	-	0.999	0.999	0.994	0.998	0.999	0.999
LOD (μg L^−1^)	67.0	-	95.3	83.8	108.9	88.7	235.1	139.2

**Table 2:** Representative examples of electrochemical sensors based on electroanalytical techniques and their applications in sensing the considered pharmaceutical residues.

Electrode	Analyte	Technique	Linear Range (mg·L^−1^)	LOD (μg·L^−1^)	Application	Ref.
AuNPs/SPE	UAC	Amperometry	3.36–33.62	2002.19	Artificial saliva	[34]
Co_3_O_4_-ERGO/SPE	UAC	DPV	0.84–84.06	252.17	Artificial saliva	[35]
Cu-BTC/CPE	UAC; DA	DPV	0.08–100.87; 0.0095–94.82	33.62 5.69	Dopamine injection and urine	[36]
GP-PEDOT: PSS/SPCE	SAL	CV	1.20–131.62	299.14	Syrup AdSV and tablets	[37]
WS_2_/AC/GCE	SAL	DPV	0.24–50.26	124.44	Human urine	[38]
Hf.WO_3_/CPE	PC; SAL	SWV	/	28.72 138.80	Pharmaceutical formulation, human urine	[39]
nanoporous gold microdisc arrays	SAL	LSV	250–2000	2530	Pharmaceutical formulation	[40]
nano gold particles modified indium tin oxide (NGITO) electrode	SAL	SWV	0.05–2.0	0.075	Pharmaceutical formulation and human biological fluids	[41]
MWCNT film coated GCE	SAL	SWV	0.19–2.4	47.8	Pharmaceutical formulation	[42]
BDD	SAL	AdSV	4.15–83	1210	Pharmaceutical formulation	[43]
oxMWCNTs/UPPy/GCE	SMX	DPV	0.50–2.76	104.60	Milk	[44]
Silver-filled MWCNT nanocomposite	SMX	DPV	0.013–17.7	2.53	Pharmaceutical formulation and human urine	[45]
SPCE	SMX	DPV	1.67–24.4	501.4	Water	[46]
CNT paste	SMX	DPV	0.35–30	100	Pharmaceutical formulation	[47]
MWCNT-Nafion modified GCE	SMX	DPV	12.6–2530	2530	Pharmaceutical formulation and human urine	[48]
BDD	SMX	SWV	1.5–15.5	291	Pharmaceutical formulation	[49]
Composite material modified with AgNP, immobilized on 3D ABS support	SMX	DPV	2.5–12.7	253	Tap water, synthetic urine, drug, cow milk, breast milk, goat milk, and honey	[28]
GO/ZnO nanocomposite modified electrode	SMX	DPV	0.025–0.38	7.32	Waste water	[50]
GCE modified with a nanocomposite prepared from graphitic carbon nitride and ZnO	SMX	DPV	0.005–280	1.67	Spiked human blood serum samples	[51]
SPCE modified with Ce(III)-doped CuO nanocomposite	SMX	DPV	7.60–91,000	2.53	Biological and drug samples	[52]
graphene-modified GCE	SMX	DPV	0.0023–7.3	0.69	Pharmaceutical formulation	[53]
SPCE	SMX	DPV	0.05–0.6	15	Water	[23]
carbon fiber paper electrode	TMP	SWV	14.5–580	18.87	Fish	[27]
CuPh/PC/GCE	TMP	SWAdASV	0.12–0.33, 0.44–1.78	198.48	River water	[54]
SPCE modified with MWCN decorated with Prussian blue nanocubes	SMX; TMP	DPV	0.25–2.5 0.03–2.9	9.6 17.4	Human urine	[21]
BDD	SMX; TMP	DPV	1.0–10 0.2–2.0	0.0036 0.0039	Pharmaceutical formulation	[55]
paper-based fully printed electrochemical sensor with reduced graphene nanoribbons	SMX; TMP	DPV	0.25–2.5 0.29–2.9	22.8 11.6	Water	[56]
Paraffin composite electrode based on MWCNT modified with SbNP	SMX; TMP	DPV	0.1–0.7 0.1–0.7	6.1 9.0	Water	[57]
rGO-AgNP/GCE	SMX; TMP	DPV	0.25–2.53; 0.30–2.96	151.97 118.50	Synthetic wastewater	[58]
GR-ZnO/GCE	SMX; TMP	DPV	0.25–10.13, 10.13–43.06; 0.30–2.96, 2.96–50.36	101.31 88.87	Tap water, lake water, urine, and human serum	[59]
MoO_2_/GCE	SMX; TMP	DPV	2.53–25.33; 0.59–5.92	36.47 37.62	/	[60]
SPCE	SAL; SMX; TMP	DPV	0.27–2.47; 0.29–11.10; 0.47–9.04	83.76 88.65 139.23	River water	This work
**/**	SAL	GC-MS/MS	0.25–2	10	Human urine	[61]
**/**	SAL	HPLC	0.5–3.0	21	Tablet	[62]
**/**	TMP, SMX	HPLC	0.5–20.0, 1.0–25.0	410, 820	Human plasma	[63]
**/**	SMX, TMP	SPE–tandem mass spectrometry	12–400, 1.2–40	470, 60	Human serum	[64]

AdSV: adsorptive stripping voltammetry; BDD: boron-doped diamond; Co_3_O_4_-ERGO: Co_3_O_4_ nanoparticles–electrochemically reduced graphene oxide; CNT: carbon nanotube; CPE: carbon paste electrode; Cu-BTC: Cu-benzene-1,3,5-tricarboxylic acid; CuPh/PC: copper phthalocyanine/printex L6 carbon black; DA: dopamine; GCE: glassy carbon electrode; GP-PEDOT: PSS: graphene-poly (3, 4-ethylenedioxythiophene): poly(styrene-sulfonate); GR-ZnO: graphene–ZnO nanorods; Hf.WO_3_: hafnium-doped tungsten oxide; LSV: linear sweep voltammetry; SPCE: screen-printed carbon electrode; oxMWCNTs/Uppy: oxidized multiwalled carbon nanotubes/ultrathin overoxidized polypyrrole; rGO-AgNP: reduced graphene oxide–silver nanoparticle; SWAdASV: square-wave adsorptive anodic stripping voltammetry; WS_2_/AC: disulfite tungsten/activated carbon; SWV: square wave voltammetry. GC-MS/MS: gas chromatography–tandem mass spectrometry; HPLC: high-performance liquid chromatography; TRFIA: time-resolved fluoroimmunoassay; SPE–tandem mass spectrometry: solid-phase extraction–tandem mass spectrometry.

**Table 3:** Repeatability and reproducibility values for the simultaneous determination of SAL, SMX, and TMP by DPV in 0.1 mol L^−1^ Tris-HCl buffer (pH 7) on SPCE.

	SAL	SMX	TMP
Repeatability (RSD, %)	1.3	3.5	1.6
Reproducibility (RSD, %)	2.8	3.9	4.3

**Table 4:** Determination of SAL, SMX, and TMP analytes in spiked river water by DPV on SPCE by standard addition calibration method.

Analyte	C_spiked_ (mg L^−1^)	C_determined_ (mg L^−1^)	RSD (%)	Recovery (%)
SAL	0.96	0.94 (0.02)	1.8	98.8
SMX	1.02	1.00 (0.03)	3.3	98.8
TMP	1.19	1.16 (0.03)	2.4	97.0

## Data Availability

The data that support the findings of this study are available upon written request to the corresponding authors.

## Supplementary Materials

The following supporting information can be downloaded at: https://www.mdpi.com/article/10.3390/s25102998/s1, Figure S1: schematic representation of the oxidation mechanisms of (A) UAC, (B) SAL, (C) SMX and (D) TMP, related to the separation of the obtained differential pulse voltammetry signals. Figure S2: Individual signals of 25 mg L^−1^ UAC (A), SAL (B), SMX (C) and TMP (D) solutions measured by DPV in 0.1 mol L^−1^ Britton-Robinson buffer at different pH values. Figure S3: Evaluation of buffer effect on simultaneous DPV measurements of the analytes at a concentration of 25 mg L^−1^ (A) in a pH range from 6 to 8 and the DPV plot of the most well-defined and separated signals achieved using Tris-HCl buffer at pH 7 (B). Figure S4: Simultaneous DPV responses of the different analytes at increasing concentrations. [UAC]: 3.4 to 16.3 mg L^−1^ [SAL]: 1.7 to 6.0 mg L^−1^ [SMX]: 5.1 to 24.5 mg L^−1^ [TMP]: 5.7 to 20.5 mg L^−1^. Figure S5: Nyquist plot obtained from EIS analysis of the bare screen-printed carbon electrode used for the simultaneous determination of SAL, SMX, TMP. Figure S6: Interference evaluation for the simultaneous determination of salbutamol, sulfamethoxazole, and trimethoprim at a fixed concentration of 1.5 mg/L (within the linear range) in the presence of varying concentrations of interferents: (A) ascorbic acid, (B) paracetamol, and (C) dopamine. Interferent-to-analyte ratios tested were 0:1, 1:3, 1:1, and 3:1. Normalized signal area plots illustrate the influence of interferent concentration on the obtained DPV analyte signal. Figure S7: Long-term stability evaluation of salbutamol, sulfamethoxazole, and trimethoprim over 7 days. (A) Peak area variations of individually prepared solutions of salbutamol, sulfamethoxazole, and trimethoprim (25 mg/L each), measured on days 1, 4, and 7. (B) Peak area variations of a mixed solution containing salbutamol, sulfamethoxazole, and trimethoprim (8.3 mg/L each), measured simultaneously on days 1, 4, and 7.
