# Supplementary material for: Voltammetric Determination of Salbutamol, Sulfamethoxazole, and Trimethoprim as Anthropogenic Impact Indicators Using Commercial Screen-Printed Electrodes
Source: Sensors (Basel). 2025 May 9;25(10):2998. doi: 10.3390/s25102998 (PMC12115316; doi:10.3390/s25102998)
Supplement: Supplementary file 1 [file sensors-25-02998-s001.zip › sensors-3592618-supplementary.pdf]

SUPPLEMENTARY INFORMATION

Voltammetric determination of salbutamol, sulfamethoxazole and trimethoprim as anthropogenic impact indicators using commercial screen-printed electrodes

J. Huang, J. Bastos-Arrieta*, N. Serrano, J.M. Díaz-Cruz*

Department of Chemical Engineering and Analytical Chemistry, Universitat de Barcelona (UB), Martí i Franqués 1-11, 08028 Barcelona, Spain, e-mail: josemanuel.diaz@ub.edu / julio.bastos@ub.edu

Water Research Institute (IdRA), University of Barcelona (UB), Martí i Franquès 1-11, 08028-Barcelona, Spain

*Corresponding author to whom correspondence should be addressed.

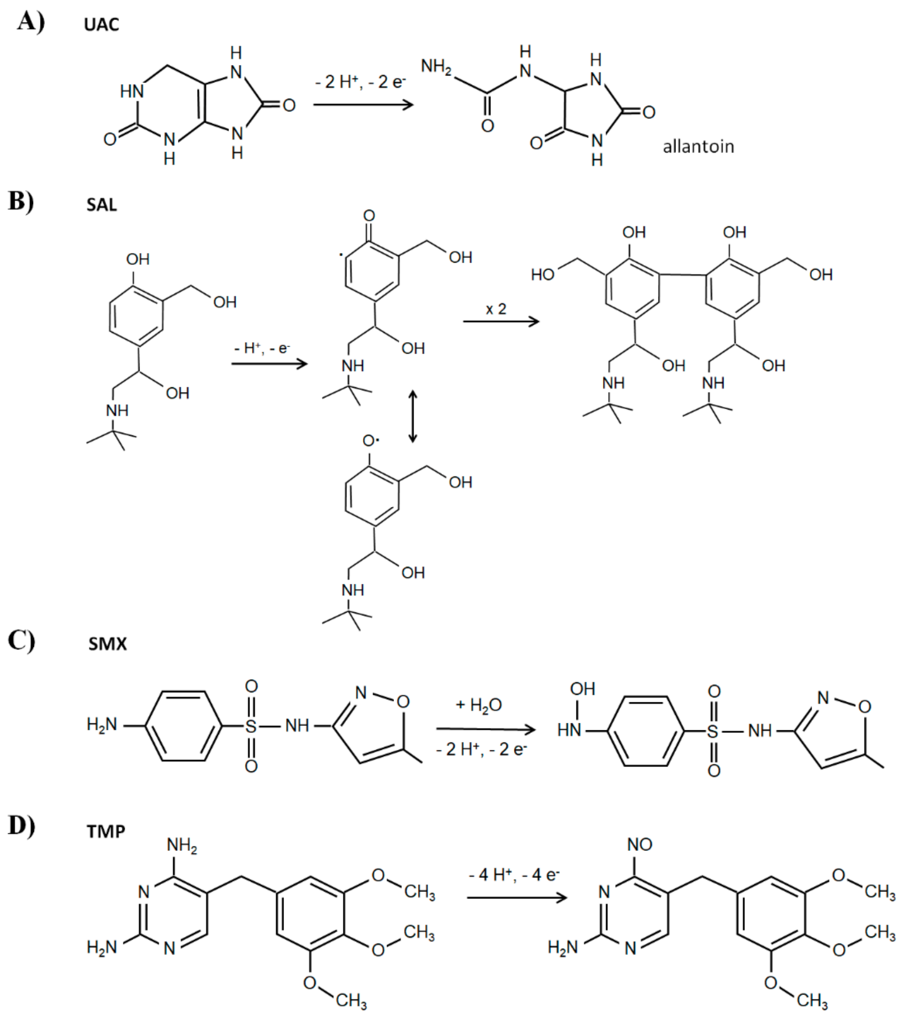

Figure S1. schematic representation of the oxidation mechanisms of A) UAC, B) SAL, C) SMX and D) TMP, related to the separation of the obtained differential pulse voltammetry signals

SUPPLEMENTARY INFORMATION

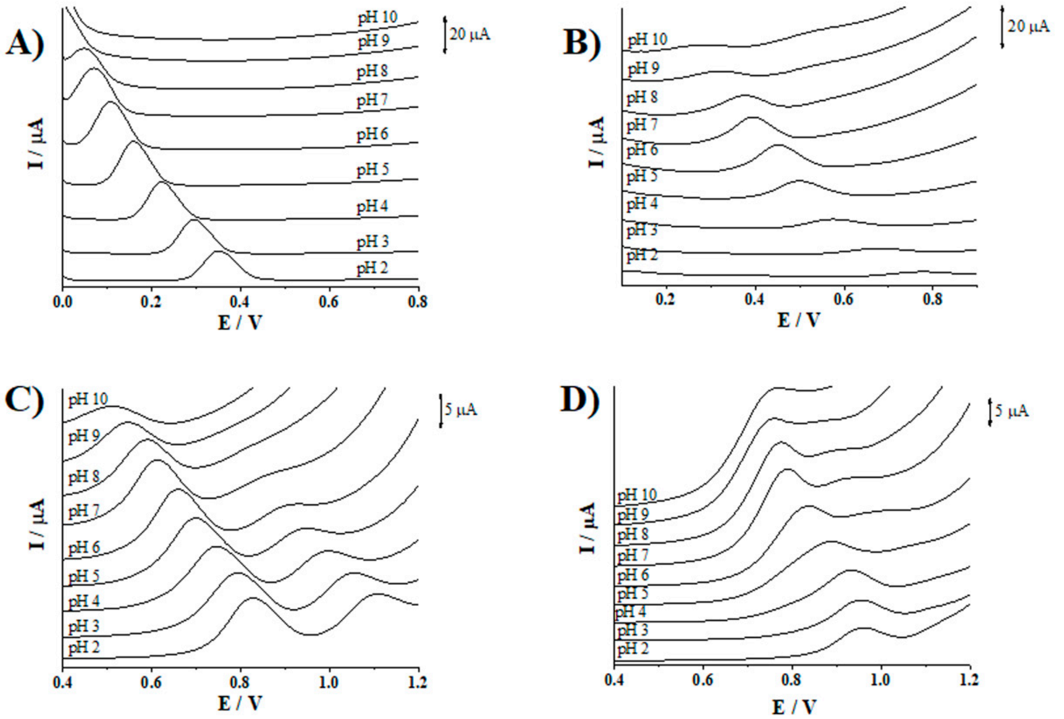

Figure S2. Individual signals of 25 mg L^{-1} UAC (A), SAL (B), SMX (C) and TMP (D) solutions measured by DPV in 0.1 mol L^{-1} Britton-Robinson buffer at different pH values.

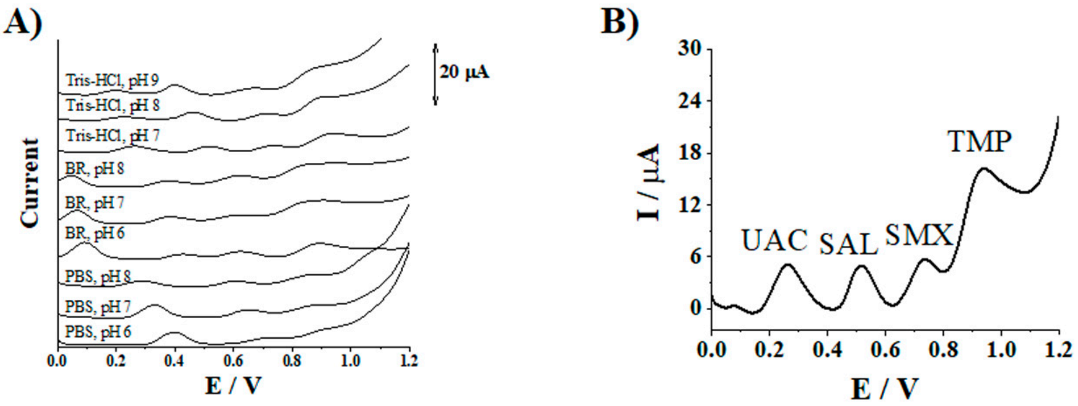

Figure S3. Evaluation of buffer effect on simultaneous DPV measurements of the analytes at a concentration of 25 mg L^{-1} (A) in a pH range from 6 to 8 and the DPV plot of the most well-defined and separated signals achieved using Tris-HCl buffer at pH 7 (B).

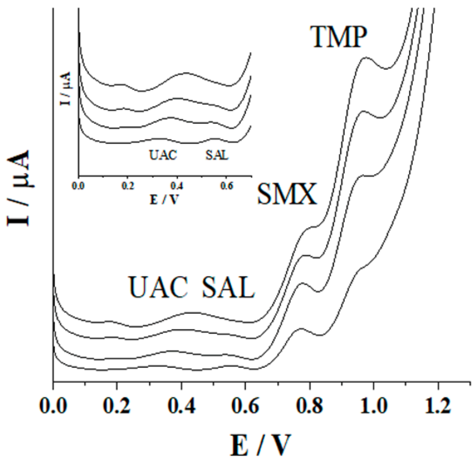

Figure S4. Simultaneous DPV responses of the different analytes at increasing concentrations. [UAC]: 3.4 to 16.3 mg L^{-1} [SAL]:1.7 to 6.0 mg L^{-1} [SMX]: 5.1 to 24.5 mg L^{-1} [TMP]: 5.7 to 20.5 mg L^{-1}

SUPPLEMENTARY INFORMATION

Electrochemical Impedance Spectroscopy (EIS)

A multi Autolab/M204) potentiostat/galvanostat (Metrohm, Herisau, Switzerland) was used to perform EIS characterization, in which Nyquist diagrams were recorded in a solution containing 5 mM $[Fe(CN)_6]^{-3/-4}$ and 0.1 mol L^{-1} KCl. The studied frequency ranged from 0.1 Hz to 100 kHz with an alternating current (AC) amplitude of 10 mV.

Nyquist plot presented in Figure S5, shows a charge transfer resistance (Rct) close to 2100 **Ω**. This value is somehow high for a conductive surface. Nevertheless, it proved to be suitable for the simultaneous determination of SAL, SMX and TMP. In addition, this value can be improved by modifying SPCE surface with different strategies, like chemical activation, drop-casting of nanomaterials and conductive polymers which may offer enhanced electrocatalytic parameters and response towards these analytes using the same voltametric methodology. Despite further possible improvements, the versatility, cost effectiveness and portability of the bare SPCE result an advantageous added value consideration

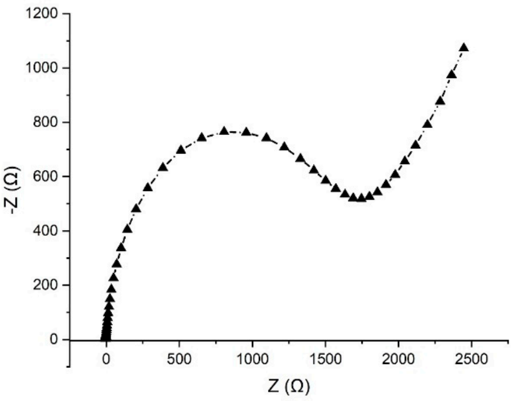

Figure S5. Nyquist plot obtained from EIS analysis of the bare screen-printed carbon electrode used for the simultaneous determination of SAL, SMX, TMP.

SUPPLEMENTARY INFORMATION

Interference Assay

The effect of other organic compounds as interferents of the developed voltammetric method was carried out using ascorbic acid, paracetamol and dopamine as model interferent analytes. These present different charges (negative, neutral and positive) at the pH of the analysis. The evaluation was performed considering different ratios of interferent/analyte as shown in Figure S6. In all cases, SAL is extremely affected by the presence of the interferents, maybe due to its chemical structure, which makes it more pH dependent. On the other hand, SMX and TMP seem not to be affected by the presence of ascorbic acid or paracetamol, but Dopamine affects greatly its signal. Reducing the effect of these interferents could be accomplished by the surface modification of the SPCE. Nevertheless, the suitability of the method for environmental analysis is supported by the standard addition results described in the manuscript.

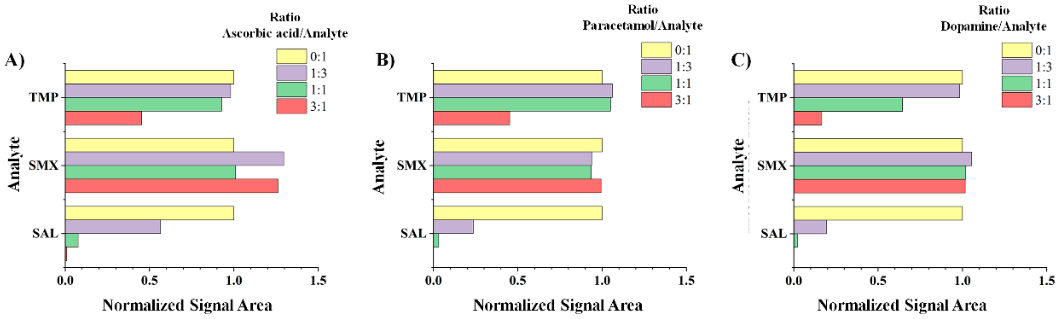

Figure S6. Interference evaluation for the simultaneous determination of salbutamol, sulfamethoxazole, and trimethoprim at a fixed concentration of 1.5 mg/L (within the linear range) in the presence of varying concentrations of interferents: (A) ascorbic acid, (B) paracetamol, and (C) dopamine. Interferent-to-analyte ratios tested were 0:1, 1:3, 1:1, and 3:1. Normalized signal area plots illustrate the influence of interferent concentration on the obtained DPV analyte signal.

SUPPLEMENTARY INFORMATION

Stability and reproducibility assays:

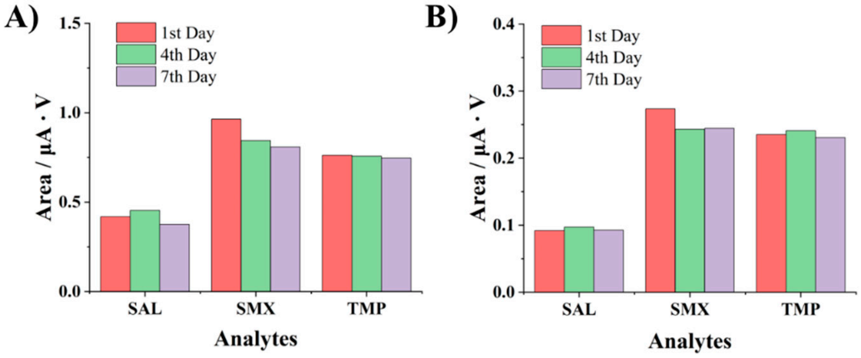

Figure S7. Long-term stability evaluation of salbutamol, sulfamethoxazole, and trimethoprim over 7 days. (A) Peak area variations of individually prepared solutions of salbutamol, sulfamethoxazole, and trimethoprim (25 mg/L each), measured on days 1, 4, and 7. (B) Peak area variations of a mixed solution containing salbutamol, sulfamethoxazole, and trimethoprim (8.3 mg/L each), measured simultaneously on days 1, 4, and 7.

For the individual determination (Figure S7A), SAL and SMX exhibited noticeable signal variability over the 7 days, with relative standard deviations (RSDs) of 9.51% and 9.32%, respectively. This suggests potential instability, likely due to degradation, adsorption, or solubility-related factors during storage. In contrast, TMP demonstrated good stability with an RSD of only 1.10%, indicating minimal change across the tested time points.

Regarding the simultaneous determination of the analytes in Figure S7B, all three analytes displayed more consistent peak areas. The RSDs for SAL, SMX, and TMP decreased to 2.93%, 6.77%, and 2.21%, respectively. Notably, the substantial reduction in SAL's RSD suggests improved stability in the mixed system. This may be attributed to buffering or synergistic effects within the multicomponent solution that mitigate degradation or signal drift.

Overall, the results support the method's applicability and reliability for multi-component long-term analysis.
